# Glycemic variability associated with gamma secretase inhibitor therapy in a type 1 diabetic patient undergoing treatment for desmoid tumor: a case report

**DOI:** 10.3389/fendo.2026.1800553

**Published:** 2026-05-01

**Authors:** Jean Carlos Ramos Cardona, Renee Morecroft, Suzanne Martinez, David Anez

**Affiliations:** Internal Medicine, Hospital Corperation of America (HCA) Florida Orange Park Hospital, Orange Park, FL, United States

**Keywords:** desmoid tumor, diabetes mellitus, gamma secretase inhibitor, glycemic variability, type 1 diabetes mellitus

## Abstract

**Introduction:**

Desmoid tumors, also known as aggressive fibromatosis, are rare, locally invasive soft tissue neoplasms characterized by clonal fibroblastic proliferation without metastatic potential. Gamma secretase inhibitors (GSIs) are currently being investigated for use in desmoid tumor therapy. This therapy targets the Notch pathway which is associated with this tumor’s pathogenesis can affect tumor growth, glucose metabolism and insulin sensitivity. Current data on this association is limited and has only been described *in vitro* and *in vivo* mouse studies. Phase 3 of the DeFi trial (N = 69), demonstrates glycosuria in approximately 5 participants but data on adverse effects are not currently available for the phase 2 RINGSIDE trial. This case uniquely describes GSI associated hyperglycemia in a type 1 diabetic patient enrolled in the RINGSIDE trial for treatment of a left breast desmoid tumor.

**Case description:**

A 31-year-old female with previously well controlled Type 1 Diabetes Mellitus and left breast desmoid tumor presented with new onset glycemic variability after enrollment in a clinical trial involving a gamma secretase inhibitor. While on therapy, her clinical course was complicated by fluctuating glycemic control with recurrent diabetic ketoacidosis and hypoglycemic episodes requiring frequent adjustments to her insulin therapy. Her glycemic control stabilized after being disenrolled from the clinical trial.

**Conclusion:**

We present a real-world case describing the association of GSIs with glycemic instability. Prior studies (*in vitro* or *in vivo* mice models) have demonstrated the role of the Notch pathway in glycemic control; however, the data is limited especially in the realm of clinical practice. It also highlights the complexities of managing glycemic control in diabetic patients undergoing GSI therapy and emphasizes the importance of close endocrine follow up as well as integrated, multidisciplinary care.

## Introduction

Desmoid tumors are rare fibroproliferative soft tissue neoplasms characterized by locally aggressive growth and a high propensity for recurrence, without metastatic potential. They affect approximately 3–5 individuals per million annually and are associated with a high risk of local recurrence (1, 2). Gamma secretase inhibitors (GSI), such as nirogacestat and AL102, are emerging therapies for desmoid tumors that target the Notch signaling pathway, a key driver in tumor pathogenesis (1, 2). Clinical trials, including the phase 3 DeFi trial and the ongoing RINGSIDE trial, have demonstrated promising results, with improvements in progression-free survival and symptom control (3). However, GSIs are associated with a range of adverse effects, many of which are attributed to Notch pathway inhibition (4). Hyperglycemia has emerged as a notable adverse effect and is hypothesized to result from disruption of Notch-mediated regulation of glucose metabolism and insulin sensitivity (4). To our knowledge, there are limited published clinical reports describing glycemic variability associated with GSI therapy in patients with type 1 diabetes (T1D). Here, we present a real-world case observed during participation in the RINGSIDE clinical trial for desmoid tumor therapy.

## Case description

**Figure 1:**
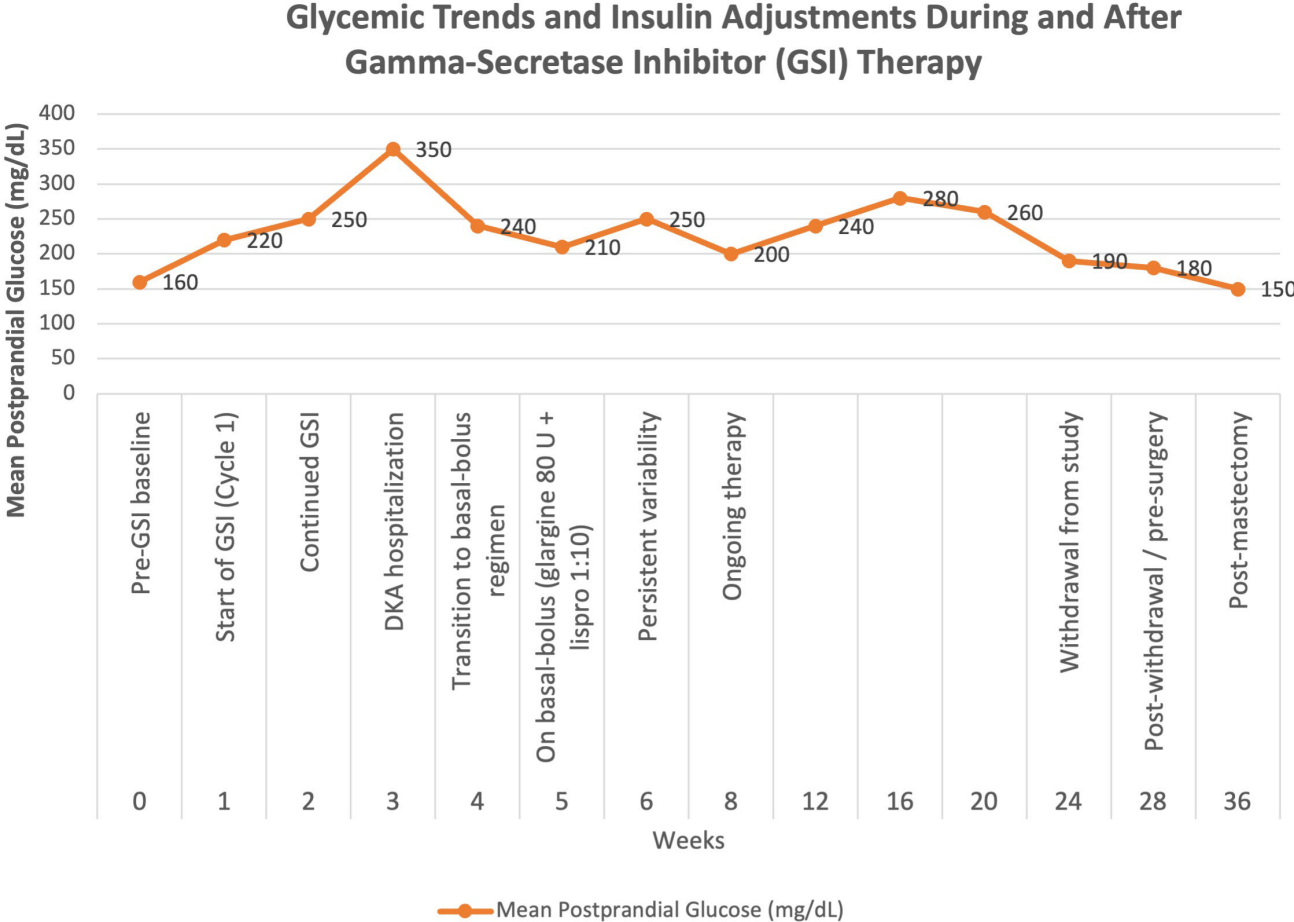
Weekly postprandial glucose mean levels over time in a patient with T1D undergoing gamma-secretase inhibitor (GSI) therapy for desmoid tumor. Hyperglycemia developed within the first week of treatment, peaked during week 3 with diabetic ketoacidosis, and gradually improved after GSI discontinuation and post-mastectomy recovery.

**Figure 2:**
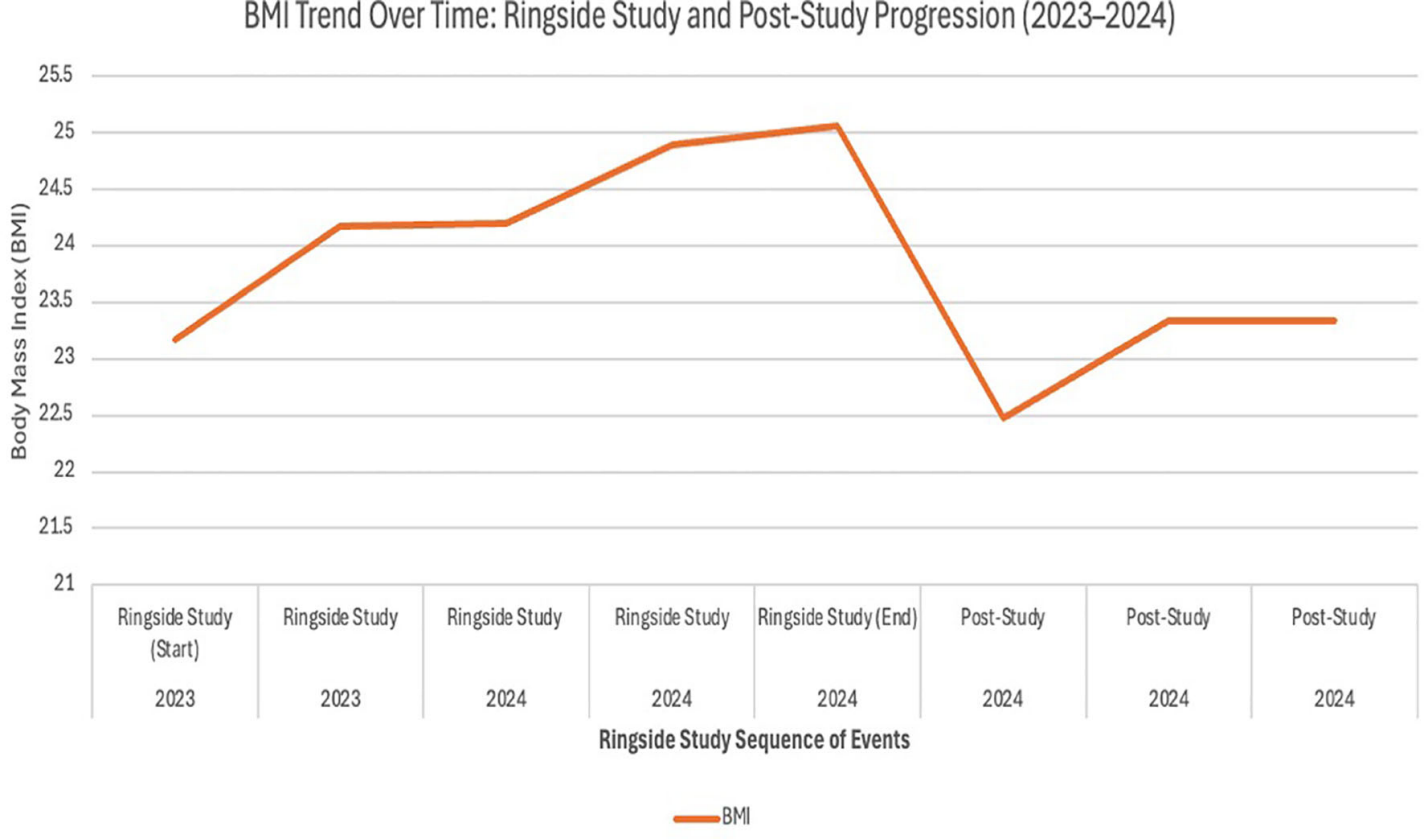
BMI trend over time: ringside study and post-study progression (2023–2024). This graph illustrates the progression of Body Mass Index (BMI) measurements collected from 2023 through 2024. The initial data points represent the start of the Ringside Study in 2023, followed by a final measurement at the study’s conclusion in 2024. Subsequent values reflect post-study BMI changes, highlighting an overall upward trend over time.

A 31-year-old female with a history of well controlled T1D diagnosed 22 years prior, thyroid nodules, and left breast desmoid tumor presented to our endocrinology clinic for evaluation of worsening glycemic control. Three years prior to presentation, she noted rapid enlargement of a previously stable left breast mass, which was confirmed on biopsy to be a β-catenin–positive desmoid tumor. She subsequently enrolled in the RINGSIDE clinical trial investigating GSI for desmoid tumor treatment. Hyperglycemia was first documented during cycle 1 of therapy, with a blood glucose level of 256 mg/dL. Changes in mean glucose levels and BMI throughout the clinical course are summarized in **Figures 1** and **2**.

Shortly thereafter, she was hospitalized for diabetic ketoacidosis, despite adherence to her premixed 70/30 insulin regimen, which she had been using due to cost-related limitations following loss of insurance coverage. No identifiable precipitating factors including infection or pregnancy were identified. While the temporal association raised concern for treatment-related glycemic dysregulation, alternative factors including suboptimal insulin regimen and access related barriers were also present. Her glycemic control remained inadequate, with blood glucose levels ranging from 180–300 mg/dL despite escalation of her 70/30 insulin dose from 18 to 30 units twice daily. The patient declined continuous glucose monitoring and insulin pump therapy.

She subsequently transitioned to a basal-bolus insulin protocol consisting of glargine 80 units in the morning and lispro at a 1:10 carbohydrate ratio. Although partial improvement was observed, she continued to experience significant glycemic variability, including episodes of symptomatic hypoglycemia. Her basal insulin was gradually reduced to 70 and then 65 units, with adjustment of prandial insulin dosing. Laboratory A1C levels fluctuated between 7.9% and 8.3% over this period. Interpretation of this case is limited by several confounding factors, including transitions in insulin regimen, cost-related barriers to optimal diabetes management, lack of continuous glucose monitoring data, and physiologic stress related to malignancy and treatment.

## Timeline

## Diagnostic assessment

Evaluation was based on clinical assessment, serial blood glucose monitoring, hemoglobin A1c measurements, and review of oncologic history and treatment exposure. A detailed timeline of the patient’s clinical course is provided in **Table 1**. Hyperglycemia was first noted during cycle 1 of GSI therapy, with a blood glucose level of 256 mg/dL, followed by hospitalization for diabetic ketoacidosis. Subsequent monitoring showed persistent hyperglycemia despite escalating insulin doses. Hemoglobin A1c values fluctuated between 8.3% and 7.9%.

**Table 1:** Glycemic trends and insulin adjustments during and after gamma-secretase inhibitor (GSI) therapy.

Date	Phase	Key clinical findings	Blood Glucose trends	A1C	Insulin regimen
2023	Pre-GSI baseline	Prior to trial enrollment	100–150 mg/dL	7.1	On Novolin 70/30 →18 units twice a day
2023	GSI Initiation	DKA (no infection/pregnancy trigger)	180–300 mg/dL	–	On Novolin 70/30 → increased to 30 units twice a day
2023	Initial Endocrine Visit/Trial Ongoing	Poor control after starting gamma secretase inhibitor	180–300 mg/dL	7.2	Novolin 70/30 → increased to 70U AM/60U PM
2023	Follow-up	High variability, poor diet	70–464 mg/dL	–	Switched to Basal-Bolus: Toujeo 80U daily + Humalog (1:10 carb ratio)
2024	Follow-up	Hypoglycemia + non-adherence	Fluctuating	8.3	Toujeo ↓ to 70U, continue Humalog carb ratio
2024	Improved control	Symptomatic hypoglycemia	70–120 mg/dL (low 56)	8.2	Toujeo ↓ to 65U, Humalog adjusted to 1:9
2024	Trial discontinued	Reduced hypoglycemia	Improving	7.9	Toujeo 60U, Humalog 1:10
2024	Post-trial	Self-adjustment	Hypglycemcepisodes	–	Toujeo ↓ to 35U, reinforce Humalog adherence

Summary of glycemic trends during and after gamma-secretase inhibitor (GSI) therapy. Hyperglycemia developed soon after treatment initiation, peaking with diabetic ketoacidosis in week 3. Despite basal-bolus insulin adjustments, glucose levels remained variable during therapy. Following GSI discontinuation, glycemia stabilized and HbA1c improved to 7.3% at follow-up.

Distinguishing medication-related dysglycemia from other causes of DKA and poor glycemic control was challenging. The patient reported insulin adherence, and no precipitating factors such as infection were identified.

The diagnosis was suspected GSI-associated glycemic variability. Alternative considerations included progression of autoimmune diabetes; however the temporal relationship with GSI initiation and absence of other triggers supported a treatment-related etiology. The patient demonstrated marked glycemic variability with recurrent hyperglycemia indicating increased risk for acute metabolic complications and the need for close endocrinologic follow-up during ongoing therapy.

## Follow-up and outcomes

Despite medication adjustments and education on dietary adherence, she experienced persistent hypoglycemia, particularly at night. Her basal insulin was further decreased to 60 units, though she independently reduced it to 40 units, which led to erratic BG levels. Ultimately, she was withdrawn from the study due to tumor progression and medication intolerance with gastrointestinal adverse effects which were not amenable to symptomatic control. She subsequently underwent a left breast total mastectomy approximately one year ago. Her insulin regimen was readjusted post mastectomy to glargine 35 units daily, maintaining the lispro 1:10 ratio with overall improved glycemic control up to her currently.

## Discussion

To date, there are limited published real-world reports describing glycemic variability associated with GSI therapy. In this case, a patient with type 1 diabetes developed significant glycemic instability, including episodes of diabetic ketoacidosis and hypoglycemia, following initiation of GSI therapy for desmoid tumor treatment. Although glycemic control improved after discontinuation of therapy, this association should be interpreted with caution given the presence of multiple potential confounders.

GSIs, including agents like nirogacestat and AL102, have demonstrated potential in managing desmoid tumors—a rare form of soft-tissue tumor known for its local aggressiveness and high recurrence rate. These drugs exert their effects by targeting the Notch signaling pathway, which plays a key role in the development of these tumors. Nirogacestat blocks the proteolytic activation of the Notch receptor, thereby inhibiting Notch pathway signaling and tumor growth. AL102 is another GSI currently under investigation which inhibits gamma-secretase-mediated cleavage of the Notch receptor. AL102 and nirogacestat are currently being investigated in the RINGSIDE and DeFi clinical trials respectively (2). Unfortunately, data on the adverse effects for the RINGSIDE trial, in which our patient participated are not yet available as it is currently in phase 2 of investigation.

However, there are a few *in vitro* and *in vivo* mice model studies which demonstrate the relationship between insulin and GSI use through several complex mechanisms. One notable pathway involves their role in the cleavage of the insulin receptor. Using *in vitro* HepG2 liver-derived cells, the insulin receptor undergoes sequential cleavage by calpain 2 and gamma-secretase, a process that disrupts insulin signaling by producing soluble insulin receptor, which is linked to reduced insulin sensitivity (5). Therefore inhibiting gamma-secretase, through GSIs can prevent this cleavage, thereby potentially enhancing insulin signaling and mitigating insulin resistance. However, the effects of GSIs are not uniform across tissues. In mice models, Sparling et al. showed that selectively blocking gamma-secretase in adipocytes led to diminished insulin sensitivity in fat tissue, suggesting a decrease in glucose uptake (4). This indicates that while GSIs may enhance insulin signaling in the liver, they could simultaneously impair glucose metabolism in adipose tissue. Their impact is likely determined by the interplay between enhanced hepatic insulin action and reduced adipose glucose disposal.

Research into gamma secretase inhibition in the context of T1D, has primarily focused on its role in pancreatic beta-cell development and function. An *in vitro* study highlighted that inhibition of gamma secretase with GSI IX (N-[N-(3,5-Difluorophenacetyl-L-alanyl)]-S-phenyl-glycine t-Butyl Ester) was shown to encourage the differentiation of rat embryonic pancreatic precursor cells into insulin-producing islet-like clusters (6). Using a three-dimensional hydrogel culture system, the researchers observed a marked increase in insulin-positive cells and enhanced glucose-stimulated insulin secretion—levels comparable to those seen in mature rat islets. These findings suggest that GSIs could play a role in generating functional beta cells (6). However, the effects of GSIs are not limited to Notch signaling. Presenilin, an integral part of the gamma secretase complex, has also been implicated in insulin signaling regulation through pathways independent of gamma secretase activity (7). Presenilin inhibits insulin receptor expression resulting in decreased downstream insulin signaling (7). Inhibition of this mechanism via a GSI would result in the opposite effect. Therefore, while gamma secretase inhibition shows potential for promoting the differentiation of insulin-producing cells, its broader impact on insulin signaling and beta-cell health remains complex.

Based on the mechanisms described, GSIs contribute to glycemic variability through distinct pathways. This complex interplay likely explains the alternating episodes of hyperglycemia and hypoglycemia observed in our case, which necessitated frequent adjustments to the patient’s insulin regimen. Continuous glucose monitoring would have been valuable for tracking these fluctuations, but the patient declined this method of monitoring. Notably, the stabilization of the patient’s blood glucose levels following the discontinuation of GSI therapy further supports the likelihood of drug-induced glycemic variability. Unfortunately, there is currently no clinical trial data to confirm this association, highlighting the need for further investigation potentially in phase 3 of the RINGSIDE trial.

The predominant glycemic disturbance was significant variability rather than sustained hyperglycemia. Importantly, hypoglycemic episodes were not consistently temporally associated with insulin dose escalation, supporting a potential contribution from GSI therapy, although confounding cannot be excluded.

This report has several limitations. Continuous glucose monitoring data were not available, limiting detailed assessment of glycemic variability. Concurrent changes in the insulin regimen represent a potential confounder. A drug rechallenge was not performed. These factors limit the ability to establish a definitive causal association between gamma secretase inhibitor therapy and the observed glycemic instability.

## Patient perspective

From patient’s perspective, glycemic control was difficult while receiving cancer related therapy. Following improved glycemic control, the patient expressed satisfaction with her management and outcome.

## Data Availability

The raw data supporting the conclusions of this article will be made available by the authors, without undue reservation.
